# Factors contributing to the recognition of anxiety and depression in general practice

**DOI:** 10.1186/s12875-018-0784-8

**Published:** 2018-06-23

**Authors:** Henny Sinnema, Berend Terluin, Daniëlle Volker, Michel Wensing, Anton van Balkom

**Affiliations:** 1 Netherlands Institute of Mental Health and Addiction, Trimbos Institute, Postbox 725, 3500 AS Utrecht, The Netherlands; 2 Department of General Practice and Elderly Care Medicine, Amsterdam Public Health Research Institute, VU University Medical Centre, Van der Boechorststraat 7, 1081 BT Amsterdam, The Netherlands; 3 Universitatsklinikum Heidelberg, Im Neuenheimer Feld 130.3, 69120 Heidelberg, Germany; 4 Department of Psychiatry, VU University Medical Centre and GGZinGeest, AJ Ernststraat 887, 1081 HL Amsterdam, The Netherlands

**Keywords:** Anxiety, Depression, Recognition, Primary care, General practitioner

## Abstract

**Background:**

Adequate recognition of anxiety and depression by general practitioners (GPs) can be improved. Research on factors that are associated with recognition is limited and shows mixed results. The aim of this study was to explore which patient and GP characteristics are associated with recognition of anxiety and depression.

**Methods:**

We performed a secondary analysis on data from 444 patients who were recruited for a randomized trial. Recognition of anxiety and depression was defined in terms of information in the medical records, in patients who screened positive on the extended Kessler 10 (EK-10). A total of 10 patient and GP characteristics, measured at baseline, were tested and included in a multilevel regression model to examine their impact on recognition.

**Results:**

Patients who reported a perceived need for psychological care (OR = 2.54, 95% CI 1.60–4.03) and those with higher 4DSQ distress scores (OR = 1.03; 95% CI 1.00–1.07) were more likely to be recognized. In addition, patients’ anxiety or depression was less likely to be recognized when GPs were less confident in their abilities to identify depression (OR = 0.97; 95% CI 0.95–0.99). Patients’ age, chronic medical condition, somatisation, severity of anxiety and depression, and functional status were not associated with the recognition of anxiety and depression.

**Conclusions:**

There is room for improvement of the recognition of anxiety and depression. Quality improvement activities that focus on increasing GPs’ confidence in the ability to identify symptoms of distress, anxiety and depression, as part of care according to guidelines, may improve recognition.

**Electronic supplementary material:**

The online version of this article (10.1186/s12875-018-0784-8) contains supplementary material, which is available to authorized users.

## Background

Anxiety and depression are highly prevalent, negatively impact everyday functioning, cause great suffering, and incur high healthcare costs and costs associated with reduced productivity [1–3]. Although clinical guidelines are available [4, 5], the management of these disorders in general practice is often suboptimal. Under-recognition of anxiety and depression has been reported, although more severe symptoms may be more easily recognized [6, 7]. Adequate recognition, diagnosis and treatment of anxiety and depression may decrease the burden of disease [8]. With approximately 75% of adult patients visiting their general practitioner (GP) at least once a year in the Netherlands, the GP is in a good position to detect anxiety and depression [9]. In the Netherlands more than 75% of patients diagnosed with psychological problems are treated in general practice [10]. Studies showed a wide range of recognition rates of depression and anxiety in primary care, also depending on the method of case ascertainment and the time allowed for GPs to recognize [11–13]. Recognition rates were higher when patient medical record extraction was used over an extended period compared to cross-sectional methods. In addition, when a less specific definition of recognition was used, recognition rates were higher. However, this may also result in more false positives [14].

Characteristics of both GPs and patients influence recognition of anxiety and depression. Many patients do not acknowledge that they suffer from anxiety or depression, and may present themselves in general practice with somatic symptoms [15–18]. Both patients and GPs may prioritise physical problems if they coexist with a (hidden) depression [19]. Even when a psychiatric diagnosis is made the patient or GP may not perceive a need for treatment [20]. Furthermore, some GPs find it difficult to distinguish between ‘normal’ distress and depression requiring treatment [21]. In the presence of chronic physical health problems GPs and patients tend to normalise distress [22]. In addition, there are barriers related to the access of care (e.g. stigma, lack of information about mental health and available services) [23].

Adequate recognition, and subsequent treatment, could improve outcomes for patients. Previous studies have examined different patient and GP characteristics as possible factors associated with the recognition of depression and anxiety, showing mixed results. Factors associated with recognition include female gender, advanced age, being single, severe depression, comorbid anxiety or depression, chronic somatic co-morbidity, history of depression, having disclosed mental health problems to the GP, and positive attitudes toward help seeking [7, 14, 24–29]. A few studies also examined whether primary care physician characteristics, such as years of experience, education, special interest, knowledge and skills, were associated with recognition of depression [7, 24] and anxiety [14]. Wittchen et al. (2001) found that physicians with more than 5 years of practice experience were more likely to recognize patients with a depression. Janssen et al. (2012) and Piek et al. (2012) concluded that there were no GP characteristics associated with recognition [7, 14]. Furthermore, GPs’ attitudes likely constitute an important factor affecting the recognition of anxiety and depression [30–32].

Previous studies showed mixed results regarding factors associated with recognition and other factors are rarely studied. The factors include: (i) patient characteristics: age, married or living together, chronic medical condition, the perceived need for care, psychological symptoms and functional status, and (ii) GP characteristics: attitudes toward the management of anxiety and depression. The aim of the current study was to examine whether these factors are associated with the recognition of anxiety and depression in general practice.

## Methods

### Study design

This study is a secondary analysis of data from a cluster randomized controlled trial of tailored interventions, to improve the management of anxiety and depression in primary care (NTR1912) [33]. The aim of the trial was to determine the clinical and cost effectiveness of tailored interventions to improve compliance with guidelines for the recognition of anxiety and depression in general practice [34–37]. The trial compared training and feedback for GPs with training and feedback supplemented with a tailored intervention. Results showed that the tailored intervention resulted in increased recognition of anxiety and depression (42% versus 31%; OR = 1.60; 95% CI:1.01–2.53) [38].The identification of barriers to implementation of guidelines, the development of interventions targeting these barriers, and the application and perceived usefulness of the resulting tailored interventions have been described elsewhere [39]. The sample size was based on the primary trial objectives. The trial was approved by the medical ethics committee of the Institutions for Mental Health (METiGG; Utrecht, the Netherlands) in 2009.

### Study population

The study population included 46 GPs in 23 general practices (12 practices were randomised to the intervention condition and 11 practices to the control condition), and all patients aged 18 years or older visiting one of the participating GPs between September 2010 and June 2011. A total of 7410 patients received an information letter and an invitation to participate and were asked to complete the extended Kessler 10 (EK-10), a validated screening tool for anxiety and depressive disorder in primary care [40]. The screening was returned by 1687 patients (response rate 23%) and 766 of them (45%) screened positive and were contacted by telephone. Based on predefined exclusion criteria 158 (21%) patients were excluded. Exclusion criteria were suicidal ideation and behavior, dementia or other severe cognitive disorders, psychotic disorder, bipolar disorder, dependence on alcohol or drugs, severe unstable somatic condition, insufficient knowledge of the Dutch language, GP diagnosis of anxiety or depression or psychological treatment in the six months before the start of the study. Of the remaining 608 patients, 164 (27%) patients did not provide informed consent. Therefore, 444 patients screening positive for anxiety and depression were included in the present study. GPs were blind to which patients had entered the study. For further details on recruitment and selection we refer to the study protocol [33].

### Measures

#### Outcome measure

The outcome in the present study was GPs’ recognition of anxiety or depression, as evidenced by information that was available in the patients’ medical records, in the time period from 6 months before to 6 months after the EK-10 screening. Information that was deemed to be indicative of recognition was the presence of terms describing: (i) psychological complaints: anxiety, depression, worrying, sorrow or grief, stress, feeling down, disordered sleeping and unexplained somatic symptoms; (ii) International Classification of Primary Care-1 (ICPC-1) diagnostic codes [41] for anxiety, depression and related psychological problems i.e. acute stress, feeling anger or irritation, behaving irritably or angrily, neurasthenia, or (iii) Four-Dimensional Symptom Questionnaire (4DSQ) scores. The 4DSQ can be used to help recognize anxiety and depressive disorders. This self-report instrument can be used to distinguish between stress-related syndromes (termed ‘stress’, ‘burnout’ and ‘nervous breakdown’) and psychiatric disorders (i.e. anxiety and depressive disorders) [42]. Recognition was operationalised this way because diagnostic coding alone strongly underestimates the accuracy of the GP [14, 43]. The medical records were retrospectively searched by two researchers. They assessed 50 medical records independently and weighted kappa statistics were calculated. The kappa yielded an inter-rater agreement of 96% (weighted kappa = 0.91; 95% CI: 0.79–1.00).

#### Patient and GP related factors

The independent variables investigated included patient and GP characteristics that might be associated with the recognition of anxiety and depression. The data were collected at baseline.

Patient characteristics were age, married or living together, presence of a chronic medical condition, psychological symptoms, perceived need for psychological care and functional status. Chronic medical condition was measured with the Dutch Central Bureau of Statistics (CBS) list, a questionnaire containing 28 conditions [44]. Psychological symptoms were measured with the Four-Dimensional Symptom Questionnaire (4DSQ). The 4DSQ has four subscales relating to common psychopathology: distress, depression, anxiety and somatisation; high scores correspond to high symptom levels. Perceived need for psychological care (henceforth “need for care”) was measured with two questions after the 4DSQ: “Do you receive any help for these complaints” and “Do you need help to solve these complaints”, answered with yes/no. Functional status was measured using the World Health Organisation’s Disability Assessment Scale II (WHODAS II) which covers functional impairments in six domains over the past thirty days. The standardised total score, based on 32 items corrected for missing values was calculated [45, 46]. The domains are communication and understanding, getting around, self-care, getting along with people, life activities and participation in society. Scores range from 0 to 100; high scores indicate functional impairment.

GP characteristics were attitudes to anxiety and depression, measured with the Depression Attitude Questionnaire (DAQ) [47, 48] and with the REASON questionnaire [49]. The DAQ measures GPs’ interest in and attitudes toward depressive disorders; respondents are asked to indicate the degree to which they agree or disagree (a 7-point Likert scale in which the discrete anchor points were converted to a 0–100 scale, where 0 means strongly disagree and 100 strongly agree) with 20 statements based on their day-to-day clinical experience. The DAQ consists of 4 components: treatment attitude (high scores indicate a preference for antidepressant drugs, low scores for psychotherapy); professional unease (high scores indicate discomfort in dealing with depressed patients, perception that treating depression is unrewarding and that patients would be better off being managed by a specialist); depression malleability (high scores indicate pessimism about one’s ability to modify the course of depression) and depression identification (high scores indicate difficulty in differentiating depression from unhappiness, believes that it originates from recent misfortunes, and that there is likely to be little additional benefit beyond a GP’s own treatment. The REASON measures GPs’ attitudes to their role in the management of patients with depressive and anxiety disorders (scores can range from 1 to 7), and comprises two subscales: (i) professional comfort with and competence in care of mental health disorders (low scores indicate comfort and competence) and (ii) GPs’ concerns about problems with the health care system for management of anxiety and depression (low scores indicate concerns about difficulties).

### Statistical methods

Multiple imputation was used, creating 5 complete datasets to deal with missing values. The imputation model included recognition, condition (intervention or control), patient characteristics (gender, age, born in the Netherlands, married or living together, in paid employment, level of education, 4DSQ score, WHODAS II score, chronic medical condition, need for care, living conditions), and GP characteristics (attitude towards anxiety and depression). Missing values for recognition (*n* = 24), born in the Netherlands (*n* = 8), married or living together (*n* = 5), level of education (*n* = 4), living conditions (*n* = 3), 4DSQ score (4DSQ Distress *n* = 11, 4DSQ Depression n = 11, 4DSQ Anxiety *n* = 12, 4DSQ Somatization *n* = 30), WHODAS II score (*n* = 17), need for care (*n* = 34) were imputed.

The prediction of recognition was examined using multilevel logistic regression analyses. Logistic regression was used because of the binary character of the outcome variable (recognition yes/no). Multilevel analysis was used to account for the clustered design (i.e., patients were clustered within GPs and GPs were clustered within practices), which threatens the mutual independence of the observations [50]. Not taking into account the clustered nature of the data, results in under-estimation of standard errors and over-estimation of statistical significance. The analyses were performed using the Generalized Linear Mixed Models method as implemented in the Statistical Package for the Social Sciences (SPSS) 22 program. All analyses were conducted in the 5 imputed datasets and results were pooled using Rubin’s rules [51]. We considered 15 potential predictors of recognition, ensuring not to exceed the limit of 10% of the number of events (i.e. recognition, *n* = 160) in the sample [52]. First, potential predictors (patients and GP characteristics) were tested through trivariate analyses using recognition as dependent (outcome) variable and condition as effect modifier. The reason to take the trial intervention (condition) into account as a possible effect modifier was that the intervention might have affected the effect of certain predictors on recognition. Predictors and interaction terms with *p*-values < 0.20 were selected for inclusion in a multivariate logistic regression model. Then stepwise backward selection was used to remove non-significant (*p* > 0.05) predictors and interaction terms one-by one from the model, starting with predictors and interaction terms with the highest p-value. To assess the extent to which the predictors and interactions retained in the final model actually explained the variance in recognition, a receiver operating characteristic (ROC) analysis was conducted using the model-predicted probabilities as test variable and recognition as the outcome variable. The area under the ROC curve (AUC) ranges from 0.5 and 1.0 with larger values indicating more variance explained [53].

## Results

### Characteristics of the study population

Baseline characteristics for patients and GPs are given in Table 1. The mean age of the 444 patients was 54 years, and 69% were female. The mean age of the 46 GPs was 49 years, and 54% were men.

**Table 1:** Baseline characteristics of primary care participants (*n* = 444*) and general practitioners (*n* = 46). Values are numbers (percentages) unless stated otherwise

Patient characteristics	
Mean (SD) age (years)	54.3 (15.8)
Married or living together (*n* = 439)	294 (67.0%)
Number of chronic medical conditions^a^(range: 0–28), mean (SD)	3 (2.1)
4DSQ^b^ Distress score (range: 0–32), mean (SD) (*n* = 433)	12.2 (7.6)
4DSQ Depression score (range: 0–12), mean (SD) (n = 433)	1.7 (2.7)
4DSQ Anxiety score (range: 0–24), mean (SD) (*n* = 432)	3.0 (3.7)
4DSQ Somatisation score (range: 0–32), mean (SD) (*n* = 414)	8.4 (5.9)
Functional status^c^(*n* = 427)	23.9 (15.7)
Need for care (*n* = 410)	201 (49%)
General Practitioner characteristics	
DAQ^d^ mean score (SD)	
Treatment attitudes (range 0–100)	44.3 (7.2)
Professional unease (range 0–100)	44.9 (7.8)
Depression malleability (range 0–100)	40.1 (11.0)
Depression identification (range 0–100)	46.6 (11.1)
REASON^e^ mean score (SD)	
Professional comfort with and competence in care of mental health problems (range 1–7)	3.2 (0.4)
GPs’ concerns about problems with the health care system for treatment of anxiety and depression (range 1–7)	4.4 (0.8)

*n = 444 unless stated otherwise

^a^Chronic medical condition was measured with the Dutch Central Bureau of Statistics (CBS) list

^b^4DSQ = Four-Dimensional Symptom Questionnaire

^c^Functional status was measured with the WHODAS-II = World Health Organisation’s Disability Assessment Scale II (excluding work)

^d^DAQ: Depression Attitude Questionnaire

^e^REASON questionnaire: GPs’ attitudes to their role in the management of anxiety and depressive disorders

### The association of patient and GP characteristics with recognition

GPs recognized anxiety or depression in 160 patients (36%). Recognition rates were especially higher in patients who: were younger than 55 years, had a 4DSQ Distress score of ≥11, had a perceived need for psychological care, and a WHODAS II score of ≥21 (see Additional File 1). Table 2 shows the results of the trivariate and multivariate analyses. Ten predictors and/or their interaction terms with condition had *p*-values < 0.20 in the trivariate analyses and were entered in a multivariate model (in Table 2 indicated by a “‡” sign after the p-value). After the backward selection procedure only 4 predictors and one interaction term remained in the final model. Patients with higher 4DSQ distress scores (OR = 1.03; 95% CI 1.00–1.07) were significantly more likely to be recognized. Note that the OR of 1.03 is related to 1 point difference in the Distress score (range 0–32). To illustrate, if a person with 10 points on the Distress scale has a probability of 30% of being recognized, a person scoring 25 points has a probability of 40%. Also patients who had reported a need for care (OR = 2.54, 95% CI 1.60–4.03) were significantly more likely to be recognized. In addition, patients’ anxiety or depression was less likely to be recognized when GPs had less confidence in their abilities to identify depression (DAQ subscale depression identification; OR = 0.97; 95% CI 0.95–0.99). Married or living together was the only predictor showing an interaction with condition, suggesting that being married or living together was a predictor of recognition only in the intervention condition but not in the control condition. Actually, recognition was lowered in patients who were married or living together in the intervention condition (OR = 0.39; 95% CI 0.16–0.95). The ROC analysis showed an AUC of 0.698 (95% CI 0.645–0.751) suggesting that the amount of variance in recognition explained by the joint predictors in the final model was “poor” to “fair” [53]. Patients’ age, the presence of a chronic medical condition, the 4DSQ scores for somatisation, anxiety and depression, and patients’ functional status were not associated with the recognition of anxiety and depression. In addition the DAQ subscales treatment attitude, professional unease and depression malleability were not associated with recognition. As well as the REASON subscales professional comfort with and competence in care of mental health disorders and GPs’ concerns about problems with the health care system for management of anxiety and depression.

**Table 2:** Results of the trivariate and multivariate multilevel logistic regression analyses predicting recognition of anxiety and depression

Independent variables (predictors)	Trivariate analysis				Multivariate analysis		
	OR	95% CI	P		OR	95% CI	P
Condition (intervention/control)	1.50	0.94–2.39	0.087	‡	3.08	1.47–6.46	0.003
Age	0.98	0.96–1.00	0.060	‡			
Condition^a^Age	1.00	0.97–1.03	0.989				
Married or living together (ref. not married or living together)	1.40	0.77–2.52	0.268	‡	1.25	0.67–2.35	0.478
Condition^a^Married or living together	0.33	0.14–0.79	0.013	‡	0.39	0.16–0.95	0.039
Number of chronic medical conditions^a^	0.94	0.83–1.08	0.389				
Condition^a^Number of chronic medical conditions	1.03	0.85–1.25	0.784				
4DSQ^b^							
Distress	1.03	0.99–1.07	0.098	‡	1.03	1.00–1.07	0.024
Condition^a^Distress	1.04	0.99–1.10	0.153	‡			
Depression	1.04	0.95–1.15	0.393				
Condition^a^Depression	1.03	0.89–1.16	0.666				
Anxiety	1.02	0.94–1.10	0.615				
Condition^a^Anxiety	1.04	0.93–1.15	0.512				
Somatization	1.01	0.96–1.06	0.736				
Condition^a^Somatization	1.04	0.97–1.11	0.301				
Need for care (ref. no need for care)	2.77	1.56–4.91	0.001	‡	2.54	1.60–4.03	0.000
Condition^a^Need for care	1.18	0.49–2.85	0.719				
DAQ^c^							
Treatment Attitudes	1.02	0.97–1.06	0.508				
Condition^a^Treatment Attitudes	0.96	0.88–1.04	0.294				
Professional Unease	0.98	0.94–1.02	0.322	‡			
Condition^a^Professional Unease	1.05	0.99–1.11	0.137	‡			
Depression Malleability	0.98	0.95–1.00	0.084	‡			
Condition^a^Depression Malleability	1.03	0.99–1.07	0.150	‡			
Depression identification	0.96	0.94–0.99	0.003	‡	0.97	0.95–0.99	0.018
Condition^a^Depression identification	1.04	0.99–1.10	0.089	‡			
REASON^d^							
Comfort and competence with mental health care	0.52	0.27–1.03	0.060	‡			
Condition^a^Comfort and competence with mental health care	2.52	0.90–7.07	0.079	‡			
Concerns about difficulties with the health care system	1.26	0.85–1.88	0.253	‡			
Condition^a^Concerns about difficulties with the health care system	0.67	0.38–1.18	0.170	‡			
Functional status^e^	1.00	0.98–1.02	0.662	‡			
Condition^a^Functional status	1.03	1.00–1.05	0.055	‡			

Reference category: No recognition; OR = odds ratio; 95% CI = 95% confidence interval; P = p-value, ‡ variables entered in the multivariate model

^a^Interaction term

^b^4DSQ Four-Dimensional Symptom Questionnaire

^c^DAQ Depression Attitude Questionnaire

^d^REASON questionnaire: GPs’ attitudes to their role in the management of anxiety and depressive disorders

^e^Functional status was measured with the WHODAS-II World Health Organisation’s Disability Assessment Scale II

## Discussion

### Main findings

The results of this study indicate that patients with a perceived need for psychological care and those with high distress were more likely to be recognized by their GP as having anxiety or depression. This may not come as a big surprise. After all, it is very likely that patients who felt a need for care were more willing to disclose their mental health problems and express their need for care in the doctor’s office, thereby obviously increasing the likelihood of subsequent recognition. Similarly, high distress is experienced as “having a difficult time” and this is probably close to having a need for support, advice or guidance. What is more surprising, is that high depression and anxiety were not associated with recognition after accounting for distress. It seems that the severity of psychological suffering (i.e. distress) is associated with recognition, rather than the severity of anxiety and depression. No association was found between somatization and recognition. The presentation of somatic complaints unexplained by physical illness (i.e. somatization) may hinder recognition of anxiety and depression, but this was not confirmed in our study. On the GP part, we found that GPs with confidence in their ability to identify depression were more inclined to recognize patients having anxiety or depression. Besides, GPs who had received the tailored intervention were relatively less likely to recognize anxiety and depression in patients who were married or living together. Possibly, GPs in the intervention group were more focused on the recognition of anxiety and depression particularly in high-risk groups, including people having little or no social support.

In our study the amount of variance in recognition explained by the joint factors in the final model was “poor” to “fair”. Recognition seems to be largely determined by other factors, which we can only speculate about. On the part of the GP, we can think of experience with mental health problems, perceived professional responsibilities, sensitivity to emotions, and workload. On the patient part, we might think of past mental health problems, experience with mental health issues in relatives or friends, openness to own emotions, and stigma. On the part of the context of the doctor-patient encounter, we can think of the duration and quality of the doctor-patient relationship, the time available for a consultation (time pressure), and giving priority to one complaint after presentation of various complaints in one consultation.

### Comparison with existing literature

Previous studies have identified patient and GP characteristics that are associated with the recognition or diagnosis of anxiety and depression. However, results are difficult to compare because different definitions of recognition were used, as well as different instruments for the assessment of anxiety and depression.

In our study high 4DSQ distress scores were associated with the recognition of anxiety and depression. Previous research showed that the 4DSQ distress scale turned out to be effective in detecting any depressive or anxiety disorder [54]. Remarkably, high 4DSQ scores on the subscales anxiety and depression were not associated with recognition. The finding is in line with the study of Marcus et al. (2011) [27]. Patients in this study completed four symptom severity self-report questionnaires (i.e. Penn State Worry Questionnaire, Beck Depression Inventory II, Anxiety Sensitivity Index and Mini Social Phobia Inventory) and no association was found with the detection of anxiety and depression. In contrast, other studies showed that patients with more anxiety or depressive symptoms were more likely to be recognized or diagnosed [7, 14, 24–26, 29, 55]. Although instruments used in these studies were different from our study (respectively the Beck Anxiety Inventory, the Depression Screening questionnaire, General Health Questionnaire, Center for Epidemiological Studies – Depression Scale, the Composite International Diagnostic Interview and the Patient Health Questionnaire 9), research showed that correlations between the 4DSQ scales and other symptom questionnaires were positive [42]. Furthermore, in keeping with the study of Piek et al. (2012), we found no association between the presence of chronic medical illness and recognition of anxiety and depression [7]. Coventry et al. (2011) found that in the presence of chronic physical health problems GPs and patients tend to normalise distress and depression. As a consequence, depression is less frequently recognized. In addition, Hermanns et al. (2013) concluded in a review that diagnosis and treatment of depression in people with chronic illness (i.e. diabetes) can be improved [56]. An estimated 50% of patients remain undiagnosed. Other studies, on the other hand, demonstrated a positive association between physical illnesses and recognition [25, 27]. Possibly, in these studies patients with chronic somatic diseases visited their GP more frequently and, as a consequence, were more likely to be recognized. Furthermore, in line with other studies, no association was found between age and recognition [7, 26, 27]. In contrast, a few studies found that patients of higher age were more likely to be recognized [14, 24]. An explanation for this finding might be that anxiety disorders are most common in people aged between 25 and 44 years [57]. In the study of Wittchen et al. (2001) most patients were older [24]. Finally, the finding that patients’ need for care was a significant predictor of recognition was confirmed in previous research [27].

With respect to the GP characteristics, GPs’ attitudes towards anxiety and depression and their ability to detect these disorders were rarely studied. One study examined the associations between attitudes, measured with the DAQ, and clinical behaviour, including depression identification [30]. When comparing both study populations, GPs score on the DAQ components 1, 2 and 4 are similar in our study. GPs score on component 3 ‘depression malleability’ differs significantly between both studies (GPs score in our study was 40.1 and in the study of Dowrick 24.5). In the study of Dowrick et al. (2000) GP visitors completed the General Health Questionnaire 12 (GHQ-12) before the consultation and the GP rated each patient on the severity of their psychological disturbance ranging from ‘no disorder’ to ‘severe disorder’ after the consultation. Dorwick et al. (2000) found no association between GPs’ confidence in their identification of depression and the accuracy in identifying it among their patients [30]. In contrast, our study showed that GPs having confidence in the ability to identify depression were more likely to recognize patients with anxiety or depression. Possibly, Dorwick et al. (2000) found no association because they used a less specific measurement instrument for the identification of depression, the GHQ-12 which measures general distress [30]. In our study we determined recognition in patient medical records and with several indicators.

### Strengths and limitations

A strength of this study was that we identified recognition using multiple indicators in the medical records. Using diagnostic codes alone may underestimate the accuracy of GPs’ recognition of anxiety and depression [14, 43]. In addition, data on recognition was gathered longitudinally, 6 months before and 6 months after patients completed the EK-10 and were included. Compared to cross-sectional methods, record extraction over an extended period may improve the accuracy of recognition [12, 13]. For example, because patients may express their psychological complaints in repeated consultations. However, there are inherent methodological limitations when using medical records: complexities in the definition of recognition, recording, assessment and in the exercise of clinical judgement in negotiating diagnoses. Another limitation was that a self-report questionnaire, the EK-10, was used as reference standard for including the participants in this study. Although the EK-10 is an instrument for screening for anxiety and depressive disorders in general practice, a more reliable instrument as reference standard for diagnosis would have been the Composite International Diagnostic Interview (CIDI) [58]. On the other hand, using the EK-10 can also be considered to constitute a strength of the study, providing a wide definition of anxiety and depression in keeping with the relatively non-specific, heterogeneous nature of mental health conditions in general practice.

Creating a regression model using a stepwise procedure to select independent variable in the final model, carries the risk of capitalizing on chance, i.e. the risk of inclusion of variables that coincidentally show significant associations with the outcome in a particular sample. The role of marital status in the recognition of anxiety and depression may have been a chance finding.

By design, by including only positively screened patients, our study did not provide any information on false-positive recognitions.

### Practical implications and further research

Recognition of anxiety and depression is important because of its association with appropriate treatment according to guidelines [59]. Recognition could improve when GPs have more insight in factors associated with the recognition of anxiety and depression. Quality improvement activities may focus at increasing the confidence of GPs in their ability to identify symptoms of distress, anxiety and depression. Smolders et al. (2010) showed that GPs with strong confidence in their abilities to identify depression, treated their patients more often in accordance with guidelines than GPs who had difficulties with distinguishing depression from unhappiness [60]. The use of an instrument, such as the 4DSQ may be helpful to structure the dialogue with the patient, for a better understanding of the complaints and for coming to a shared understanding of the patient’s problem [39]. In the presence of chronic physical health problems, GPs and patients have a tendency to normalise distress [18] and may prioritise physical problems [19]. Using the 4DSQ may prevent normalisation of distress. The instrument is helpful in informing the patient and GP about the distress level. A high score (≥ 21) indicates a serious problem, such as a clinically significant psychiatric disorder. Hermanns et al. (2013) concluded that “self-assessment questionnaires can dramatically improve depression detection rates. Complementing such screening with assessments of psychological distress can have an additional and complementary impact on individual self-care” [56]. When using an instrument for recognition, it is important that the instrument fits the holistic focus of patient-centred consultation models favoured by GPs [61]. Certainly, recognition alone is not effective, it has to be part of care including accurate diagnosis, follow-up, and access to evidence-based treatments [62, 63]. Patients have to be informed about their anxiety and depression, about evidence-based treatment options (including watchful waiting) and patients have to express their preferences. In case of major depressive disorders patients have to be convinced to initiate and continue treatment [63]. Furthermore, because patients with a perceived need for psychological care were more likely to be recognized, patients should be encouraged in disclosing their problems. Further research is needed into which strategies (individual- and societal level) are effective to disclose mental health problems.

In our study the focus was on factors contributing to the recognition of anxiety and depression in general practice, a small part of quality improvement in primary care. For sustainable improvements in primary mental health care integration with community engagement may contribute to a better recognition of anxiety and depression by the GP [64].

## Conclusion

Patients with a perceived need for psychological care and those with high distress were more likely to be recognized by their GP as having anxiety or depression. In addition, GPs with confidence in their ability to identify depression were more likely to recognize patients with anxiety or depression. Educational efforts should concentrate on increasing GPs’ confidence in the ability to identify symptoms of distress, anxiety and depression, as part of care according to guidelines. In addition, patients should be encouraged to disclose their mental health problems.

## Additional files

Additional file 1: Percentages of patients recognised as having depression or anxiety per potential predictor. (DOCX 37 kb)

## Abbreviations

4DSQ: Four-Dimensional Symptom Questionnaire
AUC: area under the curve
CBS: Central Bureau of Statistics
CIDI: Composite International Diagnostic Interview
DAQ: Depression Attitude Questionnaire
EK-10: extended Kessler 10
GHQ-12: General Health Questionnaire 12
GPs: general practitioners
ICPC-1: International Classification of Primary Care-1
ROC: receiver operating characteristic
SPSS: Statistical Package for the Social Sciences
WHODAS II: World Health Organisation’s Disability Assessment Scale II
