# Supplementary material for: Factors contributing to the recognition of anxiety and depression in general practice
Source: BMC Fam Pract. 2018 Jun 23;19:99. doi: 10.1186/s12875-018-0784-8 (PMC6015659; doi:10.1186/s12875-018-0784-8)
Supplement: Supplementary file 1 — Percentages of patients recognised as having depression or anxiety per potential predictor. (DOCX 37 kb) [file 12875_2018_784_MOESM1_ESM.docx]

**Additional File 1**

**Percentages of patients recognised as having depression or anxiety per potential predictor.**

|  | **Recognized (%)** |
| --- | --- |
| **Patient characteristics (n=444)** |  |
| Age ≥ 55 years | 30.9 |
| < 55 years | 41.4 |
| Married or living together yes | 34.7 |
| no | 38.6 |
| chronic medical conditions^1^ (range: 0-28) ≥ 3 | 34.6 |
| < 3 | 37.5 |
| 4DSQ^2^ Distress score (range: 0–32) ≥ 11 | 42.0 |
| < 11 | 29.6 |
| 4DSQ Depression score (range: 0–12) ≥ 1 | 40.3 |
| < 1 | 32.0 |
| 4DSQ Anxiety score (range: 0–24) ≥ 2 | 39.5 |
| < 2 | 32.0 |
| 4DSQ Somatisation score (range: 0–32) ≥ 7 | 38.4 |
| < 7 | 32.9 |
| Functional status^3^  ≥ 21 | 40.8 |
| < 21 | 30.2 |
| Need for care yes | 48.5 |
| no | 24.1 |
| **General Practitioner characteristics (n=46)** |  |
| DAQ^4^ (range 0-100) |  |
| Treatment attitudes ≥ 47 | 35.4 |
| < 47 | 37.0 |
| Professional unease ≥ 43 | 35.3 |
| < 43 | 37.1 |
| Depression malleability ≥ 38 | 35.3 |
| < 38 | 37.2 |
| Depression identification ≥ 49 | 33.0 |
| < 49 | 39.2 |
| REASON^5^ (range 1-7) |  |
| Professional comfort with and competence in care of  mental health problems ≥ 4 | 0.0 |
| < 4 | 41.1 |
| GPs' concerns about problems with the health care system  for treatment of anxiety and depression ≥ 4 | 36.2 |
| < 4 | 28.6 |

^1^ Chronic medical condition was measured with the Dutch Central Bureau of Statistics (CBS) list, ^2^ 4DSQ = Four-Dimensional Symptom Questionnaire, ^3^ Functional status was measured with the WHODAS-II = World Health Organisation’s Disability Assessment Scale II (excluding work), ^4^ DAQ: Depression Attitude Questionnaire, ^5^ REASON questionnaire: GPs' attitudes to their role in the management of anxiety and depressive disorders.
